# New Hospital for Burton-On-Trent

**Published:** 1896-01-25

**Authors:** 


### NEW HOSPITAL FOR BURTON-ON-TRENT.

The opening of the new hospital for infectious diseases cases has now taken place. The institution consists of a series of blocks, situated on a sheltered terrace on the northern extremity of the Outwood hills. The total cost, inclusive of the freehold site, is estimated at about £11,000. The plans were designed and the construction of the buildings was superintended by the borough surveyor, Mr. J. E. Swindlehurst, C.E , and the contract was carried out by Mr. Hodges, of Burton.

The buildings comprise (1) the administrative block; (2) the isolation block; (3) the scarlet fever pavilion; (4) the laundry block, mortuary, and discharging-rooms; (5), bath-rooms, &c., for discharge of patients; and (6) the entrance lodge and waiting-room. All of these are of brick, having pressed brick facing, with stone sills and dressings. The buildings are erected in separate blocks, situated at distances varying from 60 ft. to 90 ft. The administrative block is intended for the use of the hospital staff and the nurses when off duty. The building is two storeys in height, with a floor in the roof, whilst it is cellared under the front portion. This front portion contains, on the ground floor, committee-room, dispensary, matron's-room, nurses' sitting-room and dining-room, work-room and linen store, kitchen, scullery, and pantry, with the necessary lavatories and offices. All sanitary arrangements are against external walls, so as to have direct connection with the outside air. On the first floor six bed-rooms are provided for the matron and nurses, with a bath-room and lavatories. On the attic floor are situated the servants' sleeping-rooms, with a bath-room, &c. It is arranged that all the cooking shall be done and the food served out to the several wards from the centre. The scarlet fever pavilion is intended to afford accommodation for the reception of patients of either sex, but all suffering from the disease. In the centre of this block the entrance hall is situated, where the patients are received, the wards being entered from it. Fixed inspection windows look into each of the wards, and a separate room opening from each ward, and situated near the nurses' duty-room, is provided. The whole block provides for 22 beds on the regulation basis. Opening from the extreme angles of the ward, and separated by ventilated passages, detached buildings are provided for bath-rooms, all direct communication with the wards being thus cut off. The wards are ventilated by means of open fires at each end, these being provided with the "Manchester grates." The ventilation of the wards has received the most careful consideration, and the floors of the wards are laid with white deal in narrow widths, the whole being polished and stained. The isolation block is situated a convenient distance from the block last described, and occupies an elevated position on the terrace. The building consists of two equal parts divided by a cross wall, and the arrangements on one side of the wall are the counterpart of those on the other. Each half contains a large ward for three beds, with small ward for one bed, together with nurses' duty-room, kitchen, movable bath, &c. The rooms communicate with each other by means of a verandah. The building is intended to be used for the reception of cases which at the time of entry are of a doubtful character. The accommodation is intended for ten beds, the sexes being divided in the two separate blocks. The laundry block is located at some distance from the hospital building, and consists of a washhouse, drying-room, ironing-room, disinfecting house, stable, ambulance sheds, &c. The disinfecting apparatus has been removed from its temporary position and re-erected at this site. The disinfector is one of the Washington Lyons' high-pressure steam machines. The mortuary is again a detached building, and is fitted up as a post-mortem room, with lavatory attached. The drainage system throughout has been most carefully devised and carried out. An entrance lodge is situated at the point of junction of the site and Outwood Lane. The accommodation here provided is for five rooms, thus permitting for an additional male attendant to live with the lodgekeeper.

